# Risk Factors for Mucosal Involvement in Bullous Pemphigoid and the Possible Mechanism: A Review

**DOI:** 10.3389/fmed.2021.680871

**Published:** 2021-05-20

**Authors:** Xinyi Chen, Wenlin Zhao, Hongzhong Jin, Li Li

**Affiliations:** ^1^Department of Dermatology, Peking Union Medical College Hospital, Chinese Academy of Medical Sciences and Peking Union Medical College, Beijing, China; ^2^Department of Dermatology, Shunyi Maternal and Children's Hospital of Beijing Children's Hospital, Beijing, China

**Keywords:** bullous pemphigoid, mucous, autoantibody, risk factors, treatment

## Abstract

Bullous pemphigoid (BP) is the most common type of autoimmune bullous disease and is characterized by the presence of circulating anti-BP180 and/or anti-BP230 autoantibodies. Patients with BP often present with tense blisters and erythema, mainly on the trunk and limbs, but a few patients also have mucosal involvement. In this article, we discuss the fact that BP patients with mucosal involvement tend to have more serious conditions and their disease is more difficult to control. Potential risk factors for mucous involvement include earlier age at onset, drugs such as dipeptidyl peptidase-4 inhibitors, cancer, and blood/serum biomarkers, including lower eosinophil count, higher erythrocyte sedimentation rate, IgG autoantibodies against both the NH_2_- and COOH-termini of BP180, and the absence of anti-BP230 antibodies. IgA and C3 deposition at the dermo-epidermal junction may also be present. Understanding these risk factors may benefit earlier diagnosis of these patients and promote the development of novel treatments. What's more, it's helpful in deeper understanding of BP development and the relationship between BP and mucous membrane pemphigoid (MMP).

## Introduction

Bullous pemphigoid (BP) is the most common type of autoimmune bullous disease and is characterized by the presence of circulating anti-BP180 and/or anti-BP230 autoantibodies. BP has an estimated incidence in different populations worldwide of between 2.4 and 21.7 per million people per year (1). BP mainly affects older patients between 60 and 80 years of aged (2). Patients with BP often present with tense blisters and erythema, mainly on the trunk and limbs, with or without mucosal involvement. Exposure to some drugs and diseases such as psoriasis, lichen planus may trigger the disease (3). Recently, studies have demonstrated that some drugs, such as dipeptidyl peptidase-4 (DPP-4) inhibitors and inhibitors of programmed death-1 (PD-1) and its ligand (PD-L1), may increase the risk of BP (4, 5). The diagnosis of BP is based on the lesion appearance, biopsy, immunofluorescence imaging of skin samples, and serologic studies (6). The choice of therapy depends on the activity and severity of BP but mainly includes glucocorticoids and other immunosuppressants. Intravenous immunoglobulin (IVIg) may be considered when the disease is poorly controlled. The use of biologic agents such as the anti-CD20 antibody rituximab is being explored, although only a few cases have been reported to date (7).

BP-associated autoantibodies mainly target the non-collagenous 16A (NC16A) domain of BP180 and the C-terminal domain of BP230. The two most accepted hypotheses for BP pathogenesis are the complement-mediated and complement-independent pathways. The complement pathway proposes that antibody binding to antigen leads to activation of complement, aggregation of neutrophils, release of proteolytic enzymes, and formation of blisters. The complement-independent pathway proposes that antigen-antibody complexes are internalized, causing proteolysis of the basement membrane (8, 9).

Although mucosal involvement is seen in relatively few BP patients, there is no denying that it causes more suffering (10, 11). For example, when the larynx is involved, patients may complain of dysphagia, sore throat, and hoarseness (12). The BP Disease Area index (BPDAI) measures involved areas of the skin and mucous membranes separately and is used to assess overall disease severity. Interestingly, BP patients with mucosal involvement display higher BPDAI scores for both the skin and blister/erosion elements, indicating that the disease is generally more severe for patients with mucosal involvement than for those without it (12–15).

To our knowledge, there has been little or no research on which BP patients are prone to mucosal involvement or how their treatment options can be improved. Therefore, in this review, we have summarized the characteristics of BP patients with mucosal involvement in terms of demography, clinicopathological manifestations, and treatment, with the goal of identifying the risk factors for mucosal involvement. In addition, we explore the potential mechanisms underlying mucosal involvement in BP for better understanding of pathogenesis as the current knowledge is limited.

## Epidemiology and Clinical Features

Typical manifestations of BP include tense bullae and erythema predominantly located on the trunk and limbs, but lesions can be polymorphic and atypical, such as those occurring in patients with mucous involvement. Unlike mucous membrane pemphigoid (MMP), in which mucosal involvement is dominant, mucosal lesions in BP patients are observed in only about 10–20% of patients and consist mainly of less aggressive erosions and blisters in the oral mucosa (16). Other mucosal surfaces, including the laryngopharynx, nasal cavity, and genitalia, are less frequently affected (17). Some patients may have the involvement of two or more mucosal surfaces sequentially rather than simultaneously (12). Although both BP and MMP show skin and mucosal lesions, there are several ways to differentiate between them. At presentation, skin involvement is the dominant manifestation of BP, while MMP is characterized by prominent mucosal involvement. Mucosal scarring is rarely seen in BP, but blisters in MMP often heal with scars that may lead to permanent disfigurement in MMP (18). BP is generally self-limiting whereas MMP runs a chronic unremitting course (19). In addition, circulating autoantibodies are usually absent or present at low titers in patients with MMP (19). Besides the NC16A domain of BP180, other antigens in MMP include the C-terminal domain of BP180, laminin 332, p200, type VII collagen, and α6β4 integrin (20). However, the diagnosis of MMP with generalized blisters and of BP with extensive mucosal involvement remains challenging especially in patients with circulating anti-BP180 autoantibodies.

Mucosal lesions can appear before, after, or virtually simultaneously with skin lesions (17). BP can be challenging to diagnose when the patient's chief complaint is haemoptysis and a sore throat (21). The oral cavity is the most frequently involved mucous membrane and is involved in about 80–94.4% of BP patients with mucosal involvement (12, 13, 22, 23). Non-keratinized mucosal surfaces such as buccal mucosa and the soft palate are more vulnerable than keratinized structures such as the gingiva and dorsum of the tongue (12). The incidence of genital involvement ranges from 0 to 20% (12, 13, 22–24). But more frequent vulval involvement is seen in children (25). Other mucous membranes, such as the nasal, pharyngeal, and esophageal mucosae, are less commonly affected. It is worth noting that BP should be considered when a patient complains of refractory mucosal lesions. Unexpectedly, Kridin et al. concluded that refractory mucosal lesions were more commonly observed in patients with head and neck lesions, which are themselves atypically affected areas in BP (12).

Topical and systemic corticosteroids and, if necessary, other immunosuppressants are the major treatments for BP. As is the case in mucous membrane pemphigoid, topical corticosteroids can also be used to treat mucosal lesions, and mucosal involvement often improves during systemic treatment of BP. To achieve disease control, patients with mucosal involvement require higher dosages of systemic corticosteroids, and many patients may require adjuvant immunosuppressants (12). Mucosal lesions respond more slowly to conventional therapy, thus prolonging the treatment duration (26). These findings indicate that BP patients in this particular group should be identified quickly to ensure they receive adjuvant therapy early in the disease course.

## Risk Factors

### Age and Gender

There are conflicting views regarding the association of BP with mucosal involvement and both age and gender. Some retrospective cohort studies demonstrated that BP patients with mucosal lesions were significantly younger than those without, but no significant differences were seen regarding gender (12, 27). However, other studies have found that mucosal involvement does not correlate with either age or gender (13, 28). Additional univariate and multivariate analyses with larger cohorts are needed to explore these relationships.

### Drugs and Cancer

Although most BP patients have no known triggers, some studies have shown associations between particular drugs and BP. Medications commonly reported include neuroleptics and diuretics. Recently, DPP-4 inhibitors and PD-1/PD-L1 inhibitors have drawn increasing attention, although the underlying mechanisms linking them to mucosal involvement in BP are unknown (29–31). DPP-4 is a multifunctional enzyme that interacts with numerous proteins, including plasminogen, which is cleaved to plasmin. Plasmin can be detected in skin lesions and blister fluid of BP patients and plays a role in the cleavage of BP180 (32). Thus, DPP-4 inhibitors may change the development of epitopes exposed within BP180, leading to lesions (33). Patients with DPP-4 inhibitor-associated BP have more frequent mucosal involvement, and the mucous BPDAI score is higher than non-DPP-4 inhibitor-related BP (34, 35). Meanwhile, patients with mucosal involvement also have a higher incidence of treatment with DPP-4 inhibitors (12). For these patients, withdrawal of DPP-4 inhibitors greatly benefits their recovery (36). Nevertheless, another retrospective cohort study found no significant difference in the incidence of mucosal involvement between patients treated with DPP-4 inhibitors and patients not (37). Although few studies have examined the incidence of mucosal involvement in BP patients who were and were not receiving PD-1/PD-L1 inhibitors, one review concluded that mucosal involvement occurred in 15.5% of patients who were treated with PD-1/PD-L1 inhibitors (38). In addition, BP associated with neoplasms may present with mucosal involvement; indeed, this may differentiate BP associated with neoplasms from other forms of BP (39). Further work will be necessary to understand the exact mechanisms by which BP is associated with drugs and neoplasms.

### Eosinophil and Erythrocyte Sedimentation Rate

Compared with BP patients who have only skin manifestations, BP patients with normal or lower eosinophil counts have a higher incidence of mucosal involvement (12, 40). Of interest, most studies have shown that eosinophilia is a special feature of BP and that circulating eosinophil numbers are closely related to the disease severity and activity (41, 42). The incidence of peripheral blood eosinophilia is from 5 to 43%(43). Eosinophil infiltration into the superficial dermis and blister cavity is one dominant feature of the disease. Eosinophils may play multiple roles in the development of BP. For example, eosinophils secrete matrix metalloproteinase-9 (MMP-9), which cleaves BP180. For another, in the presence of BP serum, IL-5 activated eosinophils promote separation of the dermo-epidermal junction (DEJ) (44). And eosinophils are required for anti-BP180 IgE-mediated skin blistering (44). Eosinophil extracellular traps also promote the separation of the dermo-epidermal junction (44). Eotaxin and monocyte chemoattractant protein (MCP)-4 may contribute to tissue eosinophilia (44). It is observed that circulating eosinophil numbers are correlated with the extent of disease activity (45). One finding revealed a significant association between dermal eosinophilia and severity of BP (46). An elevated eosinophil count is thought to increase the risk of relapse and is a predictor of poor prognosis (47). Thus, eosinophils may be a putative therapeutic target for BP. As mentioned above, patients with mucous involvement have more serious clinical presentation. But in patients with mucosal involvement, their eosinophil counts were lower. One possible explanation is that the role of eosinophils and their mechanisms of action in skin lesions may differ from those in mucosal lesions. Another possible explanation might be that eosinophils respond to the serious disease for self-protection. Thus, eosinophil involvement may occur downstream, rather than upstream, of DEJ separation. In patients with mucosal involvement, the presence of fewer eosinophils would thus lead to a lack of protection resulting in disease progression. However, one recent study found no significant correlation between the severity of mucosal involvement and peripheral eosinophilia (46). Furthermore, erythrocyte sedimentation rate (ESR) values are generally higher for BP patients with mucous involvement than for those without, but, to date, no similar associations have been demonstrated for other hematologic biomarkers, such as white blood cell (WBC) count, platelet (PLT) count, and serum albumin level (12).

### Anti-BP180/BP230 Antibodies

As mentioned above, anti-BP180 and anti-BP230 autoantibodies are the two main pathogenic antibodies in BP. To date, no significant associations have been detected between anti-BP180 antibodies and mucosal involvement, with respect to either the antibody positivity rate or antibody titers (13, 14, 46, 48). However, it is noteworthy that in patients with mucosal involvement, the values of anti-BP180 antibody levels correlate only with the erythema/urticaria BPDAI score in patients with mucosal involvement (13). Another study found that levels of BP180-specific IgG1, IgG2, IgG3, and IgG4, as measured by enzyme-linked immunosorbent assay, were higher in patients with mucosal involvement than those without (11). The majority of patients with mucosal involvement have IgG autoantibodies against both the NH_2_- and COOH-termini of the BP180 extracellular domain, whereas most patients with skin lesions alone have antibodies against only the NH_2_ terminus of BP180 (49).

In terms of antibodies against BP230, it has been reported that mucosal lesions are related immunologically to the absence of anti-BP230 antibodies (13). Unlike anti-BP180 antibodies, the positive rate but not titers of anti-BP230 antibodies is lower among patients presenting mucosal involvement (14). But, one case reported that one BP patient with oral ulcers was only positive for anti-BP230 antibodies (50). Some studies have concluded that anti-BP230 antibodies are not associated with mucosal involvement (46, 48). Since BP230 is an intracytoplasmic protein, whether antibodies against BP230 arise independently or as a secondary phenomenon of epitope spreading is still uncertain. Overall, the absence of antibodies anti-BP230 antibodies may be a risk factor for mucosal involvement, but a firm conclusion cannot yet be drawn. Of note, anti-type VII collagen autoantibodies, which are the key antibodies in epidermolysis bullosa, are detected in the serum of BP patients with, but not those without, mucosal lesions (14).

### Complement and Immunoglobulin Deposition

Direct immunofluorescence (DIF) demonstrating the linear deposition of IgG and C3 at the basement membrane zone (BMZ) is a critical test in the diagnosis of BP. Of the antibody subtypes, IgG1 and IgG4 are thought to play the most important roles in the pathogenesis of BP (9). Patients suffering from mucosal lesions have a more prominent deposition of IgG, IgA, and C3 under DIF. Studies using multiple logistic regression analysis have shown that IgA and C3 deposition at the dermo-epidermal junction is a predictive marker of mucosal involvement. Moreover, the degree of C3 deposition positively correlates with mucosal involvement (27). Maurice et al. pointed out that half of BP patients with IgA antibodies presented with mucosal involvement (51). But other researchers found no correlations between IgA and the occurrence of mucosal lesions (52). Interestingly, IgA against BP180 is the major immunoglobulin subtype in MMP. And the main epitope targeted of IgA in MMP is the C-terminal of BP180 (53). Other studies have pointed out that IgE autoantibodies are strongly correlated with BP severity (54). However, there have been no studies on the relationship between IgE and mucosal involvement. Given that immunofluorescence microscopy is commonly used in clinical practice, it may be a very useful technique for predicting mucosal involvement. Considerably more work will need to be done to determine the role of IgA in BP to compensate for current knowledge.

## Mechanisms of Mucosal Involvement

The two major antigens BP180 and BP230 are components of type I hemidesmosomes of the basement membrane zone. BP180, also known as collagen XVII (COL17), is a hemidesmosomal transmembrane protein, and its NC16A domain is the main target of the autoantibodies. As noted above, anti-BP180 antibody titers correlate with disease severity in BP (55). BP230 is an intracellular protein of the plakin family and its C-terminal domain is the major antigenic target (56). The relationship between anti-BP230 antibodies and disease severity in BP is not clearly established (57, 58). But some studies have found that anti-BP230 IgG titers have prognostic value (54, 59). Autoantibodies bind to BP180 and BP230 at the dermo-epidermal junction, leading to complement activation, recruitment of neutrophils, and the release of proteases through the complement-dependent pathway, or to internalization of complexes through the complement-independent pathway, culminating in the destruction of hemidesmosomes (8).

Because BP mainly affects the skin and mucous manifestations are relatively rare, it will be important to determine whether the autoantibody-targeted BMZ of the mucosa differs from the BMZ in skin. BP180 mRNA and protein expression levels are higher in oral keratinocytes than in skin keratinocytes, which may be a mechanism to compensate for antibody-induced depletion (60). The adhesion strength of the BMZ in oral mucosa is tighter and more difficult to disrupt than that in the skin, which may be one reason why BP patients rarely show oral lesions (60). Differences between other components of the BMZ in skin and mucosa, such as BP230 and laminin 332, will also be important topics for future research. Since the titers of anti-BP180 antibodies show no association with mucosal involvement as mentioned before, one possible explanation for mucosal involvement might be that minor trauma to the mucosal epithelium leads to antigen exposed. Moreover, saliva may function directly on the mucosa, resulting in DEJ separation. Previous studies have shown that IgA in saliva may be a diagnostic marker for MMP, similar to serum IgA in MMP (61). Whether autoantibodies exist in the saliva of BP patients and, if so, whether they lead to mucosal involvement, remains to be determined.

The finding that differential BP180 epitope recognition may be associated with different clinical presentations in BP raises the possibility that patients with mucosal involvement may produce autoantibodies targeting different sites on BP180 or BP230 (62). Kirtschig et al. found that most BP patients with oral involvement had higher titers of BP180 antibodies than patients without oral lesions, as detected using the indirect immunofluorescent technique, indicating that skins and mucosa may express different epitopes and these two kinds of patients produce autoantibodies to different epitopes (28). In support of this possibility, Hofmann et al. found that the majority of BP patients with mucous involvement had circulating antibodies against not only the NH2- terminus but also the COOH-terminus of the BP180 extracellular domain (49). Kamaguchi et al. also found that antibody pathogenicity was enhanced in the presence of both anti-NH2- and anti-COOH-terminal antibodies (60). Of note, the C-terminal of BP180 is a major antigen in MMP. Overall, these findings support the hypothesis that the presence of antibodies against the NC16A domain of BP180 accounts for skin lesions, while the presence of antibodies against another BP180 epitopes, such as that at the C-teminus, accounts for mucosal lesions. BP and MMP may thus be two subtypes on a disease spectrum, depending on the dominant or first-exposed epitope. Furthermore, non-inflammatory BP, which may be due to autoantibodies against epitopes outside of BP180 NC16A, is associated with the use of DPP-4 inhibitors, which can also increase the risk of mucosal involvement. Based on these findings, it is reasonable to speculate that some triggers of BP may induce the release of autoantibodies against BP180 epitopes other than NC16A. And the presence of anti-BP230 antibodies brings several particular clinical features of BP (63). However, the aforementioned studies have demonstrated that patients who lacked anti-BP230 antibodies were prone to mucosal lesions (13). The mechanism by which this occurs remains to be explained.

Taking these findings into consideration, we can suggest that one hit, for example trauma or administration of DDP-4 inhibitors, causes exposure of certain BP180 epitopes other than the NC16A domain in the mucosa. IgA is a crucial autoantibody and may enhance pathogenicity. As a result, antigen–autoantibody binding increases complement deposition and recruits eosinophils. Eosinophils then release eotaxin and other cytokines and secrete MMP-9, promoting DEJ separation. Two pivotal steps in this process are the exposure of different epitopes and the production of IgA with unclear functions. Complement deposition and the recruitment of eosinophils are just two of the many possible outcomes of antigen–autoantibody binding.

## Conclusion

**Figure 1:**
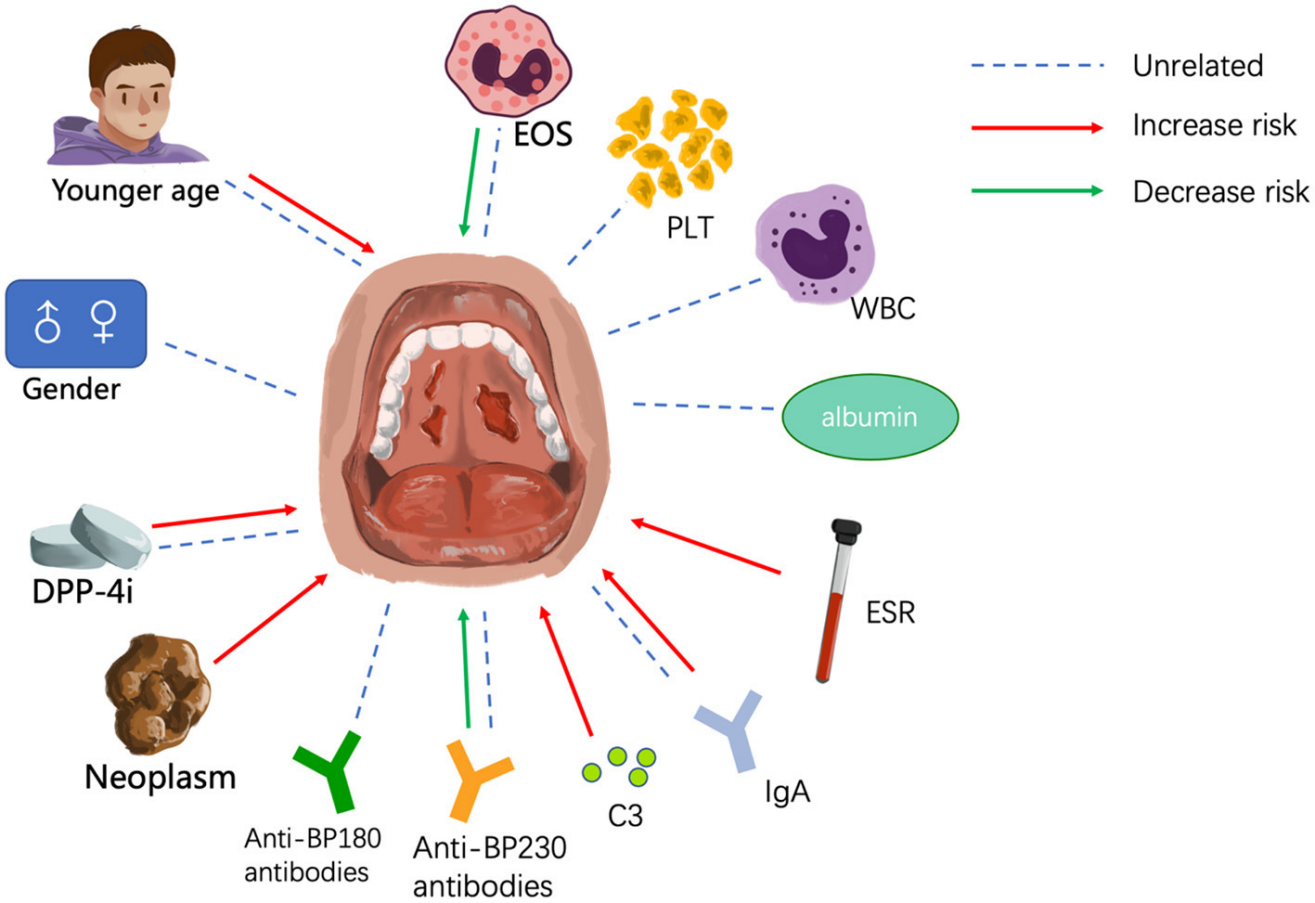
Factors that may influence mucosal involvement. EOS, eosinophil; PLT, platelet; WBC, white blood cell; ESR, erythrocyte sedimentation rate.

Although only a few BP patients present with involvement of the mucosa, such as the oral and nasal cavities, it is essential to identify these patients and administer proper treatment as promptly as possible. Here, we have summarized the clinical features and possible risk factors of mucosal involvement. BP patients with mucosal lesions tend to be younger, have more severe disease, and are less sensitive to conventional treatment than patients without mucosal involvement, which underscores the need for early diagnosis and effective therapy. Among the risk factors are earlier age of onset and treatment with DPP-4 inhibitors, and blood biomarkers of mucosal involvement include lower eosinophil count, higher erythrocyte sedimentation rate, the presence of IgG against both the NH_2_- and COOH-termini of BP180, the absence of anti-BP230 antibodies, and elevated IgA and C3 deposition at the dermo-epidermal junction (Figure 1). The details are summarized in Table 1. But these studies have mainly centered around clinical features and laboratory examinations to identify risk factors for mucosal involvement. As a result, more extensive cohort studies and case-control studies with larger sample sizes are needed to rule out interfering factors and identify the genuine risk factors. To date, the mechanism underlying mucosal involvement remains unknown. Findings to date suggest a role for distinct BP180 epitopes and IgA in promoting DEJ separation, which provides deeper insight into the relationship between MMP and BP. It is possible that MMP and BP are two subtypes on a disease spectrum and that the sequence of antigen exposure and the nature of the dominant antigen dictate the clinical manifestations. Many questions remain, and further studies will be needed to understand the role of IgA in BP, to elucidate how the BMZ in mucosa and skin may differ in other ways, to determine how cytokines and other immune-related molecules may differ between BP patients with or without mucosal involvement, to investigate the autoantibodies present in saliva, and to elucidate whether the mechanism of bullae formation in the mucosa of BP patients differs from that of MMP patients. Answering these questions will undoubtedly lead to a better understanding of both BP and MMP.

**Table 1:** Risk factors associated with mucosal involvement.

	**Risk factors for mucous involvement**	**Increase risk**	**Decrease risk**	**Unrelated**
Geographic characteristics	Younger age	(12, 27)		(13, 28)
	Gender			(12, 13, 27, 28)
Predisposing factors	Use of DPP-4 inhibitors	(34, 35)		(37)
	Neoplasm	(39)		
Routine examination	WBC count			(12)
	PLT count			(12)
	albumin			(12)
	ESR	(12)		
	EOS count		(12)	
	Peripheral eosinophilia		(12, 40)	(46)
Immunological examination	Anti-BP180 antibodies			(13, 14, 46, 48)
	Anti-BP230 antibodies		(13, 14)	(46, 48)
	C3	(27)		
	IgA	(27, 51)		(52)

## Author Contributions

XC, LL, WZ, and HJ contributed to conception and design of the review. XC wrote the first draft of the manuscript. LL, WZ, and HJ wrote sections of the manuscript. All authors contributed to manuscript revision, read, and approved the submitted version.

## Conflict of Interest

The authors declare that the research was conducted in the absence of any commercial or financial relationships that could be construed as a potential conflict of interest.
